# Comparison of SNP Calling Pipelines and NGS Platforms to Predict the Genomic Regions Harboring Candidate Genes for Nodulation in Cultivated Peanut

**DOI:** 10.3389/fgene.2020.00222

**Published:** 2020-03-24

**Authors:** Ze Peng, Zifan Zhao, Josh Paul Clevenger, Ye Chu, Dev Paudel, Peggy Ozias-Akins, Jianping Wang

**Affiliations:** ^1^Agronomy Department, University of Florida, Gainesville, FL, United States; ^2^Center for Applied Genetic Technologies, University of Georgia, Athens, GA, United States; ^3^Genetic and Genomics and Department of Horticulture, Institute of Plant Breeding, University of Georgia, Tifton, Georgia; ^4^Genetics Institute and Plant Molecular and Cellular Biology Program, University of Florida, Gainesville, FL, United States

**Keywords:** genotyping by sequencing, nodulation, peanut, RNA sequencing, single nucleotide polymorphism, SNP array, target enrichment sequencing

## Abstract

Cultivated peanut (*Arachis hypogaea* L.) forms root nodules to enable a symbiotic relationship with rhizobia for biological nitrogen fixation. To understand the genetic factors of peanut nodulation, it is fundamental to genetically map and clone the genes involved in nodulation. For genetic mapping, high throughput genotyping with a large number of polymorphic markers is critical. In this study, two sets of sister recombinant inbred lines (RILs), each containing a nodulating (Nod+) and non-nodulating (Nod-) line, and their Nod+ parental lines were extensively genotyped. Several next generation sequencing (NGS) methods including target enrichment sequencing (TES), RNA-sequencing (RNA-seq), genotyping by sequencing (GBS), and the 48K Axiom *Arachis2* SNP array, and various analysis pipelines were applied to identify single nucleotide polymorphisms (SNP) among the two sets of RILs and their parents. TES revealed the largest number of homozygous SNPs (15,947) between the original parental lines, followed by the Axiom *Arachis2* SNP array (1,887), RNA-seq (1,633), and GBS (312). Among the five SNP analysis pipelines applied, the alignment to A/B genome followed by HAPLOSWEEP revealed the largest number of homozygous SNPs and highest concordance rate (79%) with the array. A total of 222 and 1,200 homozygous SNPs were polymorphic between the Nod+ and Nod− sister RILs and between their parents, respectively. A graphical genotype map of the sister RILs was constructed with these SNPs, which demonstrated the candidate genomic regions harboring genes controlling nodulation across the whole genome. Results of this study mainly provide the pros and cons of NGS and SNP genotyping platforms for genetic mapping in peanut, and also provide potential genetic resources to narrow down the genomic regions controlling peanut nodulation, which would lay the foundation for gene cloning and improvement of nitrogen fixation in peanut.

## Introduction

Peanut (*Arachis hypogaea* L.) is one of the most important oilseed crops grown worldwide. As a legume species, peanut can form a symbiotic relationship with rhizobia to biologically fix nitrogen, thus reducing the amount of synthetic nitrogen fertilizers applied in the growing season. The symbiotic process and molecular mechanisms have been extensively studied in two model legume species *Lotus japonicas* and *Medicago truncatula*, where rhizobia enter into the host plant via an intracellular root hair pathway (Oldroyd, 2013). Many genes have been characterized in the symbiotic pathway and some rhizobial small RNA fragments were also reported to play a regulatory role (Ren et al., 2019). In peanut, rhizobia infect plants via the intercellular crack entry, which is less studied and not well understood (Peng et al., 2017a). Non-nodulating (Nod-) peanut plants, first reported by Gorbet and Burton (1979), are important materials for dissecting the genetic factors of peanut nodulation. The Nod- peanut plants were first identified in an F_3_ population from a cross between two nodulating (Nod+) genotypes 487A-4-1-2 and PI 262090 (Gorbet and Burton, 1979). Several gene inheritance models were subsequently proposed by investigating segregation ratios in populations segregating for nodulation, including the two-gene (Nigam et al., 1980), and three-gene (Dutta and Reddy, 1988; Gallo-Meagher et al., 2001) models. However, no nodulation genes have been either identified or characterized. A transcriptome study using root samples from two sets of recombinant inbred lines (RILs) with Nod+ and Nod− phenotype revealed hundreds of differentially expressed genes (DEGs) upon infection with rhizobia (Peng et al., 2017a). In addition, the same materials were morphologically and genetically characterized to initiate studies on peanut nodulation genes (Peng et al., 2018). A total of 188 simple sequence repeat (SSR) markers were used for genetic characterization, and only a few polymorphic SSRs were obtained between the RILs due to their high genetic similarity. The graphical genotype maps of the RILs were subsequently constructed showing candidate genomic regions controlling peanut nodulation and a total of 22 chromosome regions potentially related with nodulation were revealed between two sets of RILs. However, with a limited number of markers, the maps had a low resolution, which is hard for further fine mapping. With the aid of next generation sequencing (NGS) technologies, the map density could be further improved.

Peanut is an allotetraploid (2*n* = 2x = 40; AABB; ∼2.7 Gb) with two sub-genomes, A and B, derived from *A. duranensis* and *A. ipaensis*, respectively (Bertioli et al., 2016). The available reference genomes of the two diploid ancestors have made whole-genome resequencing (WGRS) an applicable approach for high throughput genotyping, which was used for genotyping a bi-parental population for high-density genetic map construction and candidate disease resistance gene identification in peanut (Agarwal et al., 2018). Each sample was sequenced at 2∼5 × coverage. However, considering the large genome size and high content of repetitive sequences in the peanut genome, WGRS may still not be the most cost-effective strategy to detect genetic variations, as the per sample cost is still high especially if high coverage is expected (Schwarze et al., 2018). Alternatively, other NGS enabled genotyping methods with reduced genome complexity can be cost-efficient for high throughput genotyping, such as RNA-sequencing (RNA-seq) (Clevenger et al., 2015; Chopra et al., 2016), genotyping by sequencing (GBS) (Tseng et al., 2016), and target-enrichment sequencing (TES) (Peng et al., 2017b), which discover genetic variations from a representative proportion of the genome. In addition, the Axiom *Arachis2* array with 47,837 SNPs can be a cost-efficient and simple method for high throughput genotyping (Clevenger et al., 2018), though it is limited to known single nucleotide polymorphisms (SNPs) only.

As the A and B genomes of peanut are highly similar with a median identity of 93.11% (Bertioli et al., 2016), it has been a big challenge to identify allelic SNPs due to the confounding effect of homoeologous SNPs between the two sub-genomes (Clevenger et al., 2015). Multiple strategies and tools have been developed to resolve this issue. One option to reduce the amount of homoeologous SNPs is to exclusively utilize uniquely mapped reads for subsequent SNP calling (Zhou et al., 2014; Peng et al., 2017b), which led to a decreased number of useful SNPs identified. Alternatively, several other methods have been developed that could use overall mapped reads for SNP calling and filter out homoeologous SNPs afterward. For example, SWEEP (Clevenger and Ozias-Akins, 2015), which utilizes homoeologous SNPs as an anchor to differentiate allelic SNPs, had been successfully applied in peanut (Clevenger et al., 2017; Pandey et al., 2017) with a validation rate of 85% through Sanger sequencing and above 95% through simulation data (Clevenger and Ozias-Akins, 2015). In addition, a machine-learning tool called SNP-ML was also developed to predict allelic SNPs with a validation rate of 75–98% (Korani et al., 2019). An improved version of SWEEP, named HAPLOSWEEP, was developed, which applies a haplotype-based method to identify allelic polymorphisms between genotypes (Clevenger et al., 2018), and it had a validation rate of 74% through genotyping by the Axiom *Arachis2* array. With these methods and tools available for the peanut community, currently no study has been performed to compare these SNP calling and filtering methods, or to compare the effects of mapping reads to the concatenated A + B genome or to A and B genomes separately (A/B).

In this study, to explore the genetic factors and genetic regions controlling nodulation in peanut, SNPs were identified between the two original Nod+ parental lines as well as between two sets of RILs. Three NGS approaches, including TES, RNA-seq, and GBS were applied and compared for SNP identification. To summarize and compare different SNP analysis methods, we have applied and compared two alignment methods (to A + B genome or to A/B genome) and various SNP calling and filtering pipelines using the sequencing data. In addition, the Axiom *Arachis2* array was also used for genotyping and served as a SNP cross-validation platform for identified SNPs. This is the first study to compare different SNP calling and filtering pipelines for various NGS data sources in peanut. Results and suggestions from this study provide insights into SNP identification and genotyping in peanut. The polymorphic genomic regions between the sister RILs revealed candidate genes controlling peanut nodulation, which will be beneficial for future genetic mapping studies.

## Materials and Methods

### Plant Materials

Two sets of RILs, E4 (Nod−) & E5 (Nod+), and E6 (Nod+) & E7 (Nod−), as well as their parental lines, PI 262090 (Nod+) and UF 487A (Nod+) were included in this study. The pedigree information of these six lines was introduced previously (Peng et al., 2017a). In brief, the two sets of RILs can be traced to two different F_6_ lines, which were deived from the cross between PI 262090 and UF 487A. They are also parental lines for two F_2_ mapping populations (E4 × E5 and E6 × E7) for genetic mapping of nodulation genes. The morphological and genetic characterizations of the RILs were previously described (Peng et al., 2018). The genomic DNA of the six genotypes was extracted by using the CTAB method (Rogers and Bendich, 1994). DNA concentration and quality were checked using agarose gel and NanoDrop.

### Probe Design, Evaluation, and Selection for Target Enrichment Sequencing

To preferably target peanut genes potentially related to nodulation, a series of genes were included for probe design. Firstly, the putative orthologous nodulation-related genes and differentially expressed genes (DEGs) upon infection of rhizobia from the previous report (Peng et al., 2017a) were included (referred to as Class I genes). For these peanut genes, the gene sequences together with 2 Kb upstream and 1 Kb downstream sequences were subjected to probe design. For the Class I genes, if there were more than four peanut genes in the same orthologous group with the nodulation-related gene in model legumes, only the top four genes (based on Blast score) were included for subsequent probe selection. Secondly, for the remaining genes that were annotated in the peanut diploid ancestors’ genomes (referred to as Class II genes), only the gene coding sequences were utilized for probe design. The probes were 120 bp long and had no overlap with each other. A total of 3,982 Class I genes were obtained from the previous transcriptome study (Peng et al., 2017a). The sequences of those genes together with the remaining 74,753 Class II gene models in the diploid ancestors’ genomes of peanut were submitted for probe design.

A probe could capture or hybridize with the DNA fragments if they share sequence similarity with each other. The genomic regions sharing sequence similarities with probes were considered as probe target regions. However, the capture efficiency would be different for target regions with different similarities. Thus, the number of target regions was investigated for the probes under different alignment identity cutoffs when they were mapped to the genome. The uniqueness and distribution of the designed probes were further evaluated.

To evaluate uniqueness of the designed probes in the genome, the probe sequences were mapped back to the diploid genomes of peanut (A + B) using Blat (Kent, 2002). A hit was defined under cutoff: e-value ≤ 1e-05; alignment identity = alignment length × percentage of identity ≥96 (120 bp × 80% = 96 bp). For easier downstream data analysis, primarily single-hit probes were selected for synthesis. A unique set of single-hit probes was obtained by using CD-HIT-EST (-c 0.8 -aL 0.8 -AL 24 -aS 0.8 -AS 24 -n 5 -T 0 -r 1) (Fu et al., 2012). All single-hit probes covering Class I genes and resistance genes annotated in the genome were selected. The remaining single-hit probes were selected to ensure an even distribution throughout the genome. To achieve this, the genome sequences were chopped into fragments using EMBOSS (Rice et al., 2000) and one probe was selected from each fragment, excluding the fragments already covered by previously selected probes.

The synthesized probes were used to capture the DNA fragments of the six genotypes. The captured DNA fragments were sequenced using the Illumina HiSeq 3000 platform (100 bp paired-end reads). The probe design, synthesis, library preparation, target enrichment, and sequencing were performed by Rapid Genomics LLC (FL, United States).

### Target Capture Efficiency and Coverage of Probes

To evaluate the probe target regions, the sequences of designed probes were aligned to the A + B genomes using Blat following the same criteria as above. The read coverage for probe target regions was assessed. In addition, the relationship between read coverage and target regions’ sequence similarities with probes was investigated, which could indicate the influence of alignment identity of probes on capture efficiency. To achieve this, different alignment identity cutoffs were applied to define a hit, including 96, 90, 84, 78, 72, 66, and 60, which correspond to 80, 75, 70, 65, 60, 55, and 50% match of probe sequences to the genome. The coordinates of those hits in the genome were extended 100 bp from both directions (in BED file), which subsequently served as target regions. Bedtools v2.24.0 (intersect) was used for assessing read coverage for target regions. The alignment files for both overall and uniquely mapped reads generated from BWA-mem (Li and Durbin, 2009), as described in section below, were used. Thus, in total seven BED files of target regions under different alignment identity cutoffs, were included for calculating on-target rate and coverage of reads.

### RNA-seq and GBS Data Sets

The RNA-seq data of these six genotypes were retrieved from the previous root transcriptome study (Peng et al., 2017a), which were deposited at the Sequence Read Archives (SRA) of the National Center for Biotechnology Information (NCBI, accession number SRP093688, BioProject PRJNA354154, and BioSample SAMN06041692-SAMN06041727). Each genotype had six cDNA libraries, for a total of 36 cDNA libraries for the six samples. In total 403,245,464 read pairs (150 bp) were included for analysis. The raw reads were trimmed with Trimmomatic (Bolger et al., 2014).

The GBS data were obtained for each genotype previously as described by Peng et al. (2017b). The restriction enzyme *Ape*KI was used for removing repetitive regions to reduce genome complexity. A total of 17,408,637 single end reads (100 bp) were obtained (data deposited in the Sequence Read Archives at NCBI under accession number of SRP154150). Raw reads from GBS data were trimmed to 64 bp using Stacks (Catchen et al., 2013).

### Read Alignment, SNP Calling and Filtering

The alignment was performed by two general methods (Table 1). In the First method, trimmed reads were mapped to A or B genome (A/B) separately, and all mapped reads were used for SNP calling (M1, M4; Table 1). In this method, a read coming from the B genome could be erroneously aligned to the A genome, since A and B genomes are quite similar (Bertioli et al., 2016). SNP calling was performed using Samtools 1.3.1 (Li et al., 2009), which was built into the SWEEP pipeline. The homoeologous SNPs generated were further utilized as an anchor for subsequent SNP filtering by using SWEEP and a machine-learning tool SNP-ML (M1). In addition, a haplotype-based genotyping tool HAPLOSWEEP (M4) was also used. So M1 was defined as alignment to A/B genome, using overall aligned reads, and SNP filtering based on SWEEP + SNP-ML and depth. M4 was defined as alignment to A/B genome, using overall aligned reads, and SNP filtering using HAPLOSWEEP (Table 1). In the Second method, trimmed reads were mapped to the *in silico* concatenated (A + B) tetraploid genome (concatenated from diploid genomes), and only uniquely mapped reads were used for subsequent analysis (M2, M3, M5; Table 1). In this method, only reads having a unique location in the tetraploid genome (according to the aligner) were used. SNP calling was performed using Samtools (Li et al., 2009). SNP filtering was performed by using conventional filtering based on read depth only (M2), SWEEP and SNP-ML (M3), or HAPLOSWEEP (M5). Thus, M2 was defined as alignment to A + B genome, using uniquely mapped reads, and SNP filtering based on depth. M3 was defined as alignment to A + B genome, using uniquely mapped reads, and SNP filtering based on SWEEP + SNP−ML and depth. M5 was defined as alignment to A + B genome, using uniquely mapped reads, and SNP filtering using HAPLOSWEEP (Table 1).

**TABLE 1:** Five different alignment and SNP filtering pipelines.

**Method ID**	**Genome reference**	**Mapped reads used**	**SNP filtering**		
			**SWEEP and SNP-ML**	**HAPLOSWEEP**	**Depth-based**
M1	A/B	Overall	Yes	No	Yes
M2	A + B	Unique	No	No	Yes
M3	A + B	Unique	Yes	No	Yes
M4	A/B	Overall	No	Yes	No
M5	A + B	Unique	No	Yes	No

*“A/B” indicates alignment to A and B genomes separately. “A + B” indicates alignment to a concatenated A and B genome.*

When analyzing TES and GBS data, Bowtie2/2.3.4.1 (default –sensitive-local) was used to align reads to A and B genomes separately (for the First method) followed by SNP filtering, which was extensively applied previously in peanut (Clevenger et al., 2017, 2018; Pandey et al., 2017). Due to a low unique mapping rate from Bowtie2, BWA-mem was used for read alignment (for the Second method), which was applied in our previous TES report (Peng et al., 2017b). Uniquely mapped reads from BWA-mem were extracted by filtering off reads with a mapping quality of zero and “XA:Z” tag. When analyzing RNA-seq data, a split aligner Tophat2.1.1 (Kim et al., 2013) was used for both the First method and the Second method, with one mismatch in the 20 bp seed and GFF files supplied (Bertioli et al., 2016). Uniquely mapped reads were extracted by using the tag “NH:i:1” and a mapping quality of “50.” “–ultimate” option was used in SWEEP with default settings for other options. For SNP-ML, “-iM peanut_RNA” was used for TES and RNA-seq data, while “-iM peanut_DNA” was used for GBS data. For HAPLOSWEEP, “HAPLOSWEEP_LONGRANGE” was used for TES and RNA-seq data (paired-end reads), and “HAPLOSWEEP” was used for GBS data (single-end reads).

Finally, SNPs called from methods M1, M2, and M3 were filtered based on read depth. A homozygous genotype was called if there were at least four reads supporting either the reference or alternate allele. A heterozygous genotype was called if there were at least two reads supporting the reference and alternate allele, respectively.

### Genotyping With the 48K Axiom *Arachis2* Array and Validating SNP Calling Results From NGS Pipelines

The DNA samples of the six parental genotypes were submitted to Affymetrix for genotyping using the recently developed 48K Axiom *Arachis2* array. The genotype calling was performed as previously described (Clevenger et al., 2018). All the SNPs (between PI 262090 and UF 487A) identified from different pipelines used were compared with genotyping results from the SNP array to identify the overlapped or shared SNPs. The polymorphic SNPs (between PI 262090 and UF 487A) identified from those pipelines were considered validated or concordant with the array if they were also polymorphic on the array and had the same genotypes with those called from the NGS methods. The validation or concordance rates for the five SNP analysis pipelines (M1–M5) were subsequently calculated.

## Results

### Probe Design and Selection for Target Enrichment Sequencing

A total of 199,673 probes were designed for the 3,982 Class I genes and 1,678,459 probes were designed for the 74,753 Class II gene models. After mapping the probe sequences to the genomes (A + B) by Blat, a total of 230,730 probes had a single unique hit (alignment identity ≥96) to the genomes. To avoid any redundancy due to genome sequence duplications, CD-HIT-EST was applied and a total of 219,850 single-hit probes remained. Among the single-hit probes, a total of 20,212 probes corresponding to 2,072 Class I genes, and 9,582 probes covering 907 resistance genes were first selected (Supplementary Table S1). In addition, 824 probes with two, three, or four hits to the genomes were also selected since they covered the genes having no single-hit probes. This led to a total of 30,081 probes being selected (Supplementary Table S1) covering the Class I and resistance genes.

To select the remaining probes covering Class II gene models, the genome sequences were chopped into 44.3 Kb fragments using EMBOSS and a total of 56,296 fragments were obtained. By excluding the 2,783 fragments that were already covered by the previously selected probes, a total of 24,922 fragments were covered by the remaining single-hit probes. Thus, three fragments were randomly excluded and one probe was selected from each of the remaining 24,919 fragments so that all the selected probes were basically evenly distributed throughout the genome. Finally, a total of 55,000 probes (Supplementary Table S1) were selected for the TES experiments.

### Summary of Sequence Statistics, Trimming and Alignment

On average, there were 14,211,850 paired-end reads (100 bp) per sample obtained from TES, 67,207,577 paired-end reads (150 bp) per sample from RNA-seq, and 2,901,440 single-end reads (100 bp) per sample from GBS (Supplementary Table S2). After trimming, 96.89% of the reads remained for TES, 88.29% for RNA-seq, and all reads remained for GBS (reads trimmed to 64 bp). When the trimmed reads were aligned to A/B (A and B genomes separately) genome, on average, the overall mapping rate was more than 96% to either A or B genome for TES, more than 53% for RNA-seq, and more than 82% for GBS. When aligning to the concatenated A + B genome, the average rate of uniquely mapped reads was 51.6% for TES, 50.26% for RNA-seq, and 19.31% for GBS (Supplementary Table S2). The low unique mapping rate for GBS was consistent with its short read (64 bp) being used for alignment, in contrast with the 100 bp read length for TES and 150 bp read length for RNA-seq. Certain level of repetitive sequences may exist in the GBS reads, which would also cause low unique mapping rate. Since A and B genomes were quite similar, shorter sequences were less likely to find a unique location when aligned to the A + B genome.

### Evaluations of Target Capture Efficiency and Coverage

**FIGURE 1:**
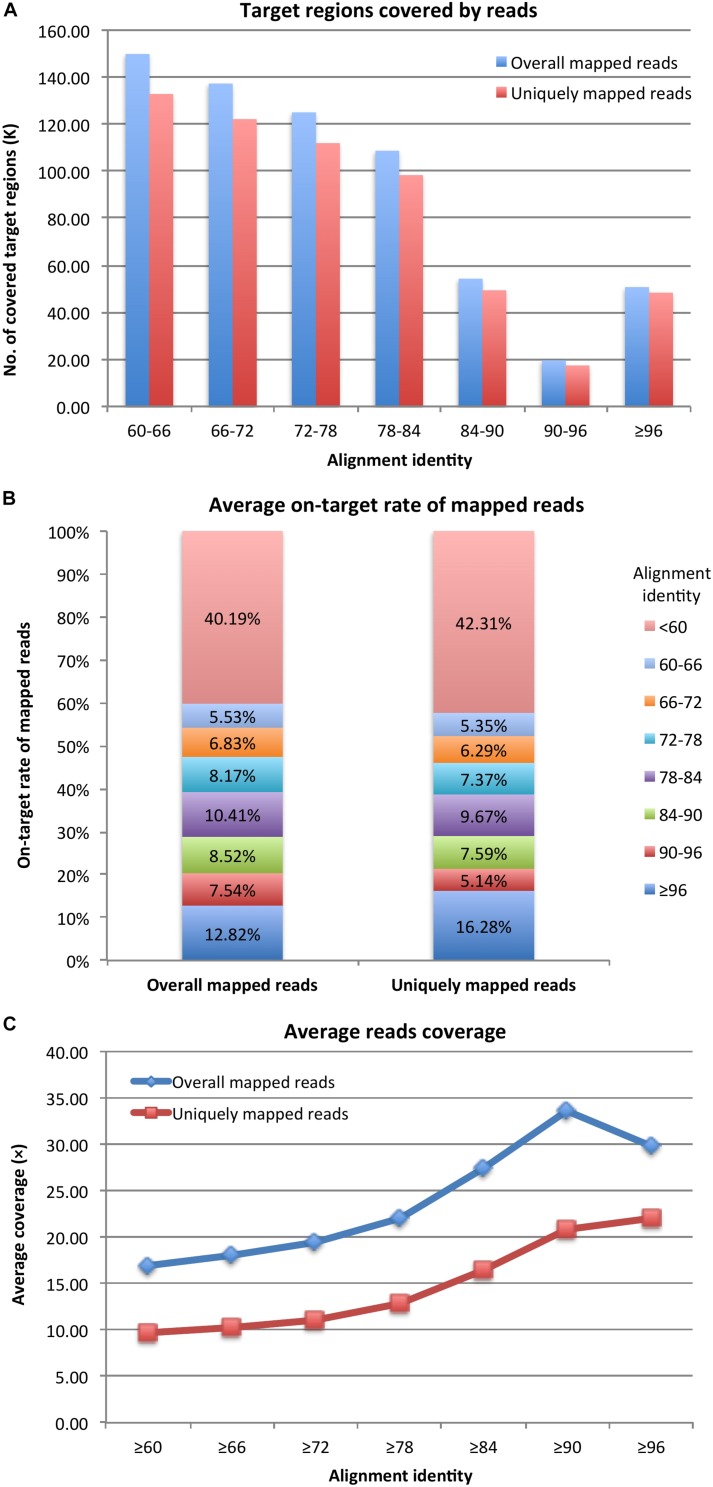
**(A)** Probe target regions, **(B)** on-target rate of mapped reads, and **(C)** reads coverage for target enrichment sequencing data.

After mapping probe sequences to the genomes, under the alignment identity cutoff of ≥96, there were 50,580 and 48,275 (91.96 and 87.77% of 55,002) probe target regions covered by reads according to overall and uniquely mapped reads, respectively (Figure 1A). By decreasing the alignment identity cutoff, more target regions were available and were covered by reads. Specifically, with an alignment identity between 60 and ∼66, there were still 149,885 and 132,787 (79.57 and 70.49% of 188,369) target regions covered by overall aligned reads and uniquely aligned reads, respectively. The average on-target rates of mapped reads to target regions with an alignment identity ≥96 were 12.82% for overall mapped reads and 16.28% for uniquely mapped reads (Figure 1B). The remaining reads were mapped to target regions with a lower alignment identity. If considering all target regions with an alignment identity ≥60, the average on-target rates were 59.81 and 57.69% (Figure 1B), respectively. Thus, probes could still capture DNA fragments even with 50% sequence similarity. However, target regions with higher sequence similarities to probes had higher read coverage (Figure 1C). Under the alignment identity cutoff of ≥96, the target regions were covered on average 29.86× and 22.05× considering overall and uniquely mapped reads, respectively. It was noteworthy that under cutoff of ≥90, corresponding to ≥75% sequence similarity, the average read coverage was 33.68× and 20.85× for overall and uniquely mapped reads, respectively (Figure 1C). The capture efficiency for cutoff 90 was comparable to that of cutoff 96. However, as the alignment identity of the probes was reduced, the average coverage of the reads captured by the probe was reduced as well. Thus, a probe could capture DNA fragments with a high and optimal efficiency if the probe sequence had ≥75% sequence similarity with the fragment sequences.

### SNP Calling for NGS Data

The alignment, SNP calling and filtering for three different NGS methods, TES, RNA-seq, and GBS data were performed using five different pipelines (Table 1). As there were more polymorphisms between PI 262090 and UF 487A, which were the two original parental lines of E4, E5, E6, and E7, the SNPs identified or validated between these two genotypes were summarized and compared among the five pipelines for the three NGS approaches (Table 2). Since these six parental genotypes were not included into the samples for developing the Axiom *Arachis2* array, the randomly overlapped SNPs between the ones identified from the five pipelines and those placed on the array were used for SNP calling cross-validation. For TES data, the largest number of SNPs (22,584) was from M2, followed by M4 (10,157), M1 (7,540), M5 (2,694), and M3 (1,283) (Table 2). However, the largest number of homozygous or genome-specific SNPs were identified from M4 (10,157), more than twice the number from M2 (4,438). Similarly, for RNA-seq data, the largest number of SNPs was from M2 (14,684), followed by M1 (1,199), M4 (901), M3 (297), and M5 (288) (Table 2). Most homozygous SNPs were also identified from M4 (901), which was higher than M2 (787). For GBS data, 278 SNPs were identified from M4, followed by M2 (171), M1 (161), M5 (15), and M3 (9). Most homozygous SNPs were called from M4 (278) and M2 (37). For all three data sources, M4 and M2 identified the largest amount of homozygous SNPs.

**TABLE 2:** Summary of SNPs between PI 262090 and UF 487A from five different methods using target enrichment sequencing RNA sequencing, and genotyping by sequencing data and concordance rate with array-overlapped SNPs.

**Data source**	**SNP analysis method**	**No. of array-overlapped/total SNPs**			**No. of concordant SNPs with the array**		
			
		**Total**	**Heterozygous**	**Homozygous**	**Total**	**Heterozygous**	**Homozygous**
TES	M1	88/7,540	86/7,316	2/224	17(19.32%)	17 (19.77%)	0
	M2	92/22,584	21/18,146	71/4,438	57(61.96%)	9 (42.86%)	48 (67.61%)
	M3	13/1,283	12/2,938	1/132	5(38.46%)	4	1
	M4	44/10,157	–	44/10,157	36(81.82%)	–	36 (81.82%)
	M5	30/2,694	–	30/2,694	23(76.67%)	–	23 (76.67%)
RNA-seq	M1	30/1,199	26/1,175	4/24	8(26.67%)	4 (15.38%)	4
	M2	108/14,684	82/13,897	26/787	33(30.56%)	10 (12.20%)	23 (88.46%)
	M3	13/297	11/285	2/18	3(23.08%)	2	1
	M4	17/901	–	17/901	14(82.35%)	–	14 (82.35%)
	M5	9/288	–	9/288	6(66.67%)	–	6 (66.67%)
GBS	M1	1/161	0/159	1/2	−	–	–
	M2	1/171	0/134	1/37	−	–	–
	M3	0/9	0/9	0/0	−	–	–
	M4	1/278	–	1/278	−	–	–
	M5	0/15	–	0/15	−	–	–

*TES indicates target enrichment sequencing; RNA-seq indicates RNA sequencing; GBS indicates genotyping by sequencing.*

### Genotyping With the Axiom *Arachis2* Array and the Concordance With NGS Methods

Genotyping using the Axiom *Arachis2* array revealed 23,060 SNP loci with high quality genotypes called for PI 262090 and UF 487A (Supplementary Table S3). Of the 23,060 SNP loci, 3,531 SNPs were polymorphic between PI 262090 and UF 487A, including 2,056 homozygous SNPs and 1,475 heterozygous SNPs (Supplementary Table S3). After comparison, the SNPs identified using HAPLOSWEEP, either using A/B or A + B as the reference, always had a higher validation rate than other SNP analysis methods based on the aforementioned overlapped SNPs (81.82% for M4, 76.67% for M5) for TES data (Table 2). The validation rate was ∼79% considering all data points. M2 had a lower concordance rate than M4 and M5, but the concordance rate for homozygous SNPs was 67.61%. All other pipelines either had too few SNPs overlapped with the array or a low concordance rate. Similarly, for the RNA-seq data, M2, M4, and M5 revealed a high concordance rate with the SNP array for homozygous SNPs (Table 2). For GBS data, there were too few SNPs from the five pipelines overlapping with those from the SNP array, therefore, they were not included for comparison.

The non-validated SNPs among the overlapped or shared SNP loci were specifically investigated. For M1, most of the non-validated SNPs proved to be polymorphic on the array. However, the genotype calls from sequence data did not match those from the array. Among the 88 overlapped SNPs, 57 (64.77%) of them were called as heterozygous SNPs from sequence data, but as homozygous SNPs from the array. This result showed that M1 was able to identify true polymorphic loci but may not assign a correct genotype due to the alignment of homoeologous reads while the sub-genome specific haplotype cannot be differentiated. In contrast, for the HAPLOSWEEP-based approaches M4 and M5, most of the genotype calls from sequence data matched those from the array (Table 2). For the remaining non-validated SNPs from M4 and M5, almost all of them proved to be polymorphic on the array, however, with either PI 262090 or UF 487A showed a heterozygous genotype, which most likely were homoeologous SNPs. Those SNPs on the array could be used as dominant markers. Similarly for M2, the most common non-validated SNP type (22 out of the 92 overlapped SNPs) was classified as a homozygous SNP from sequence data but was called as a heterozygous SNP from the array.

### Comparison of Different Platforms

**FIGURE 2:**
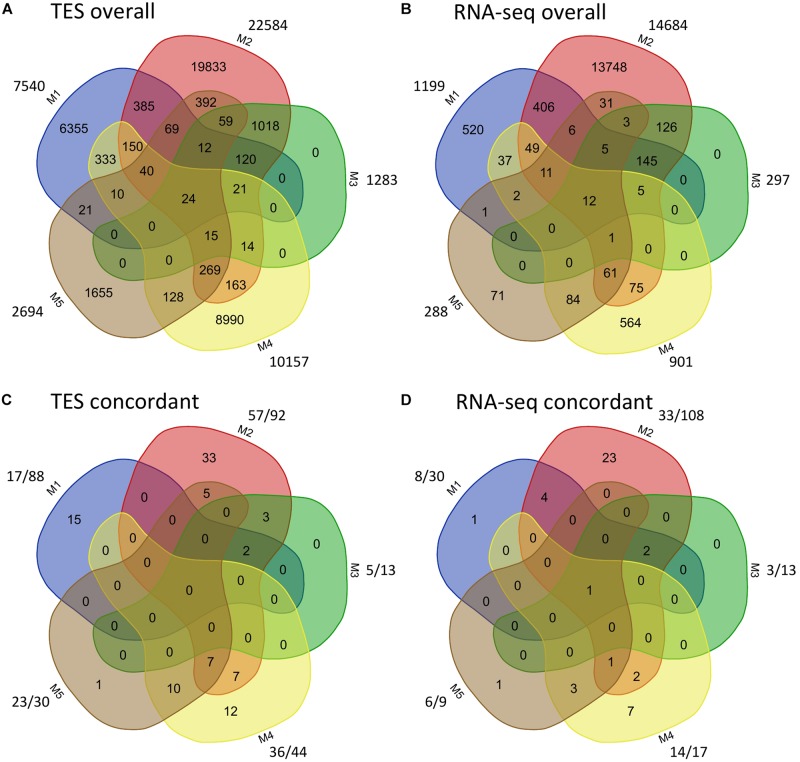
Comparison of identified and concordant SNPs among the five SNP analysis pipelines for target enrichment sequencing and RNA-sequencing data. For panels **(A,B)**, the number outside shows the total number of SNPs identified from each method. For panels **(C,D)**, the number before “/” shows the number of validated SNPs, the number after “/” shows the number of SNPs from each method that are overlapped with the Axiom *Arachis2* SNP array.

The overall called and cross-validated SNPs among the five pipelines from TES and RNA-seq were further compared (Figure 2). For both TES and RNA-seq data, a small proportion (<50%) of called SNPs were shared between M1 and M2, M2 and M4, or between M2 and M5 (Figures 2A,B). When comparing the validated SNPs, for TES data, 17 (73.91%; out of 23) of the SNPs from M5 (using A + B as the reference) were already covered by M4 (using A/B as the reference) (Figure 2C), both of which applied HAPLOSWEEP. However, only a small proportion (14 out of 57, 24.56%) of the SNPs from M2 overlapped with M4, although both revealed a high validation rate for homozygous SNPs (Figure 2C). This was also observed for RNA-seq data, in which only 4 (12.12%) out of 33 SNPs from M2 were covered by M4 (Figure 2D). These results showed that M2 and M4/M5 were able to identify different portions of true homozygous SNPs out of the existing true polymorphisms.

The performance of SNP calling and features of the three NGS methods as well as the Axiom *Arachis2* SNP array were compared (Table 3). TES revealed the highest amount of homozygous SNPs (15,947), followed by the Axiom *Arachis2* array (1,887), RNA-seq (1,633), and GBS (312) (Table 3). The per sample cost for TES was high compared to other methods, but its per sample per SNP cost was lower than RNA-seq and GBS. However, TES required pre-knowledge of DNA sequences for probe design. The lowest per sample per SNP cost came from the Axiom *Arachis2* array, which also required the least amount of analysis efforts. All three NGS methods required bioinformatics analysis of sequencing data.

**TABLE 3:** Comparison of target enrichment sequencing, RNA sequencing, genotyping by sequencing, and the Axiom *Arachis2* array.

**Items**	TES*	**RNA-seq**	**GBS**	**Axiom *Arachis2* array**
Pre-knowledge of DNA sequences	Yes	No	No	Yes
Efforts of bioinformatics analysis	High	High	High	Low
Price/sample	∼$450	∼$260	∼$35	∼$28
No. of homozygous SNPs identified	15,947	1,633	312	1,887
Per SNP per sample cost	∼$0.0282	∼$0.1592	∼$0.1122	∼$0.0148

TES indicates target enrichment sequencing; RNA-seq indicates RNA sequencing; GBS indicates genotyping by sequencing. *For 55K probes, including probe design and synthesis.

### Construction of Graphical Maps Containing Polymorphic Regions Between E4 & E5 and E6 & E7

**FIGURE 3:**
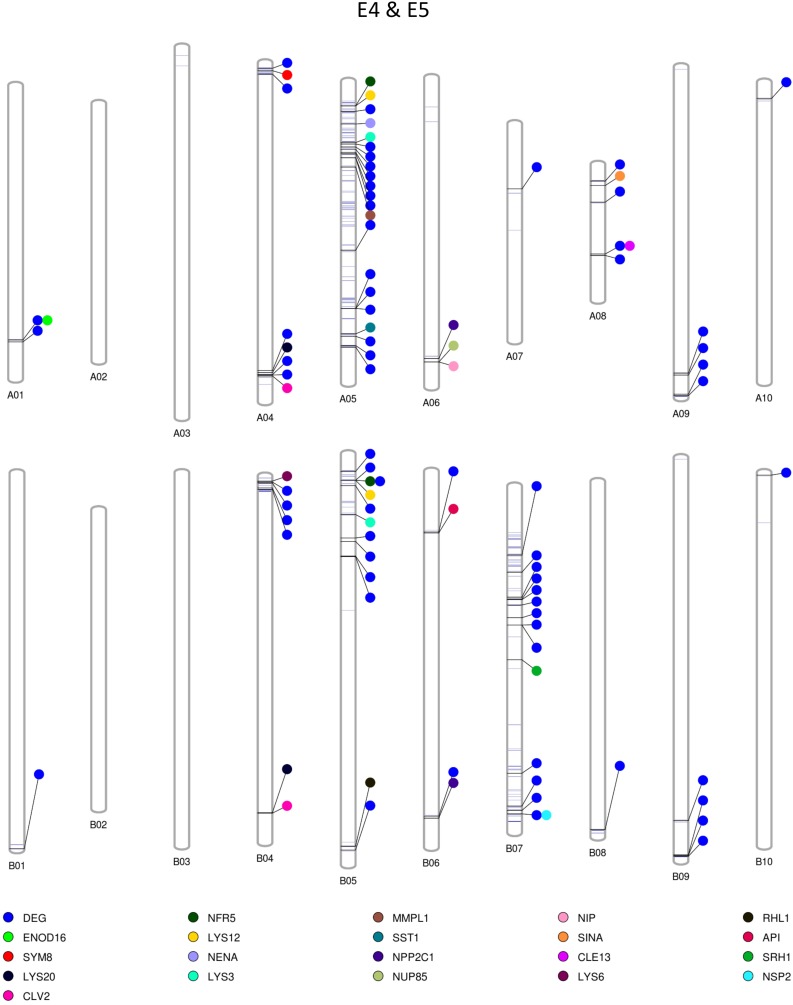
Graphical map showing polymorphic genomic regions between E4 and E5. Each line represents a homozygous SNP. Each circle represents a candidate gene.

**FIGURE 4:**
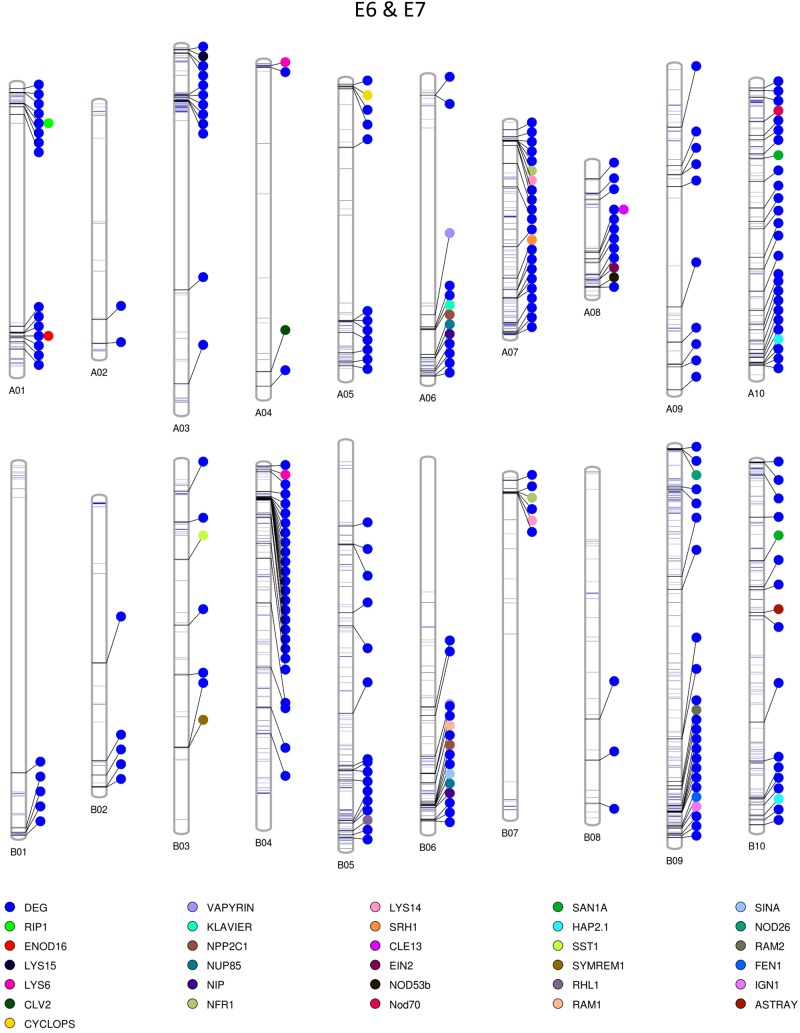
Graphical map showing polymorphic genomic regions between E6 and E7. Each line represents a homozygous SNP. Each circle represents a candidate gene.

Among the homozygous SNPs between PI 262090 and UF 487A from the Axiom *Arachis2* array, 1,859 (90.68%; out of 2,050 SNPs with high-quality genotypes) were monomorphic between E4 and E5; 1,519 (74.94%; out of 2,027 SNPs with high-quality genotypes) were monomorphic between E6 and E7. By combining the filtered SNPs identified from the three NGS methods as well as those from the Axiom *Arachis2* array, a total of 19,607 non-redundant homozygous SNPs between PI 262090 and UF 487A were obtained. Among those homozygous SNPs, a total of 222 and 1,200 were further obtained between E4 & E5 and E6 & E7, respectively, after filtering. Thus, they were placed on the graphical genotype maps (Figures 3, 4). A total of 75 polymorphic genome regions were obtained for E4 & E5, and 512 polymorphic genome regions were obtained for E6 & E7, which mostly covered and refined those genomic regions revealed by SSR markers (Peng et al., 2018) and potentially harbor genes controlling peanut nodulation. Within the 75 candidate regions of E4 & E5, there were a total of 67 DEGs and 26 putative orthologous nodulation-related genes, among which *CLE13*, *ENOD16*, *NFR5*, and *NSP2* were also DEGs (Supplementary Table S4). Within the 512 candidate regions of E6 & E7, there were a total of 217 DEGs and 39 putative orthologous nodulation-related genes, among which *CLE13*, *ENOD16*, and *RIP1* were also DEGs (Supplementary Table S4). Those genes could serve as candidate genes controlling peanut nodulation for further genetic and fine mapping.

## Discussion

In this study, we mainly focused on identifying the polymorphic regions between two pairs of sister RILs, E4 & E5, as well as E6 & E7, which are near-isogenic lines. For mapping or fine-mapping the genes controlling nodulation, polymorphic markers differentiating the near-isogenic sister lines are critical and are challenging to develop due to (1) allopolyploid nature of the cultivated peanut and (2) near-isogenic nature of the two pairs of sister lines. Therefore, in this study, we implemented multiple NGS-enabled SNP genotyping methods and SNP calling pipelines to identify reliable and sufficient number of SNP markers.

Single nucleotide polymorphisms have been extensively used for genotyping due to several favorable features such as abundance and high throughput. With the advancement of research in peanut genomics and genetics, especially the advent of reference genomes (Bertioli et al., 2016) and SNP arrays (Clevenger et al., 2017, 2018; Pandey et al., 2017), more choices of SNP genotyping became available for the peanut research community. For genetic mapping studies, WGRS approach can theoretically provide the highest resolution of marker densities. However, for crop species like peanut with a large genome size (∼2.7 Gb), it would still be costly, to have enough sequencing data to meet the requirement of coverage and depth for accurate SNP identification. Alternatively, numerous approaches, such as TES, RNA-seq, and GBS, which reduce the genome complexity by sequencing a partial genome, may be more cost-effective while still able to provide a decent number of markers. In addition, the Axiom *Arachis2* array (Clevenger et al., 2018) is another choice, which involves the least computational analysis efforts. This study utilized six peanut samples to compare SNP identification using sequencing data from different high throughput genotyping methods, TES, RNA-seq, GBS, as well as SNP array. This comparison between the different high throughput genotyping platforms provided an insight into the performance and the number of useful markers that can be generated from each platform. In the past few years, SNP marker development in allotetraploid peanut with highly identical sub-genomes used to be slow due to the presence of homoeologous SNPs (Clevenger et al., 2017). However, with the availability of tools such as SWEEP and HAPLOSWEEP, great progress has been made, which will greatly benefit the whole peanut research community. In addition to these tools, multiple analysis pipelines have also been applied for SNP identification. With so many pipeline options available, a comparison of them was needed to provide a better idea of how they differ from each other and which one outperformed the rest. Current research intended to answer these questions by applying different alignment, SNP calling and filtering methods with different sequencing approaches for SNP identification. Furthermore, the resulting SNPs revealed the polymorphic genomic regions between the sister RILs, which can narrow down the candidate regions harboring genes controlling peanut nodulation, and likely facilitate future genetic mapping and fine mapping of nodulation genes in peanut.

### Target Enrichment Sequencing

Unlike RNA-seq and GBS, which focus on genic regions or restriction site-surrounding regions, TES was able to focus on genes or genomic regions of interest. In this approach, the DNA fragments captured by custom-designed probes based on sequence homology were sequenced. Researchers can preferably design probes covering genes of interest. TES was firstly applied in peanut by using probes designed from expressed sequence tags as the sequence source for probe design (Peng et al., 2017b). In the current study, the reference genomes of the two diploid ancestors of cultivated peanut were used for probe design. In order to target symbiosis related and disease resistance related genes in peanut, a total of 20,212 probes were designed to cover all the putative nodulation-related genes and 9,582 probes to cover resistance genes. The remaining ∼24K probes were selected for an even distribution throughout the genome. Therefore, the overall density of the probes was ∼49 Kb/probe given the peanut genome size of 2.7 Gb. Out of the 78,574 peanut gene models, 26,653 (33.9%) of them were tagged by this probe set. This set of TES probes would be useful for not only mapping the genes related to nodulation or disease resistance, but also for genome association analysis of any traits considering the probe density and coverage.

During the probe selection process, single-hit probes were preferably selected, which led to the average unique mapping rate of the five samples to be 51.60%, much higher than our previous report (22.55%; Peng et al., 2017b). In addition, 91.96% of the target regions of current probe set was covered by reads with an average depth of 29.86×, which was also much higher than our previous report (average depth <20 × considering 90% of target regions; Peng et al., 2017b). Thus, utilization of the unique hit of probes in the genome is critical to improve the rate of uniquely mapped reads and depth of sequences captured by the probe set. Based on our data, probes can be very efficient in capturing DNA fragments when they have at least 75% sequence similarity with the target fragments (Figure 1C). Therefore, when applying TES, we should be aware that off-target capturing would be common specifically for the species with closely related genomes or duplicated regions in the genome.

### Comparison of Different NGS Approaches and the Axiom *Arachis2* Array

The three NGS data sources and the Axiom *Arachis2* array identified different numbers of SNPs between PI 262090 and UF 487A. Considering only the homozygous SNPs, TES identified the largest number of SNPs, followed by the SNP array, RNA-seq, and GBS (Table 3). This could be explained from several perspectives. Firstly, as TES is focused on genomic sequences, more polymorphisms are expected than that from RNA-seq representing the conserved transcribed gene regions. The low number of SNPs from GBS could be explained by the low coverage of sequencing data obtained. As there were only 2,056 homozygous SNPs between PI 262090 and UF 487A obtained from the SNP array, and even fewer SNPs for E4 & E5 and E6 & E7, the Axiom *Arachis2* SNP array may not be suitable for future genotyping of the mapping populations with E4 & E5 and E6 & E7 as the parental lines. TES can be considered as a choice due to the large number of polymorphisms discovered. Moreover, the sample per SNP cost of TES is still low compared to the other NGS methods and comparable to that of the Axiom *Arachis2* SNP array.

### Comparison of Different SNP Analysis Pipelines

From the results of comparisons between the five different pipelines for peanut SNP calling, several points can be drawn. (1) The concordance rate of heterozygous SNPs was always low between TES and RNA-seq. This could be caused by false positive SNPs derived from the misalignment of reads from homoeologous regions on the genome. (2) The alignment to A/B genome followed by SWEEP and SNP-ML filtering (M1) revealed a considerably smaller proportion of homozygous SNPs than the alignment to A + B genome followed by traditional filtering (M2), and HAPLOSWEEP approaches M4, and M5. As SWEEP was not able to differentiate haplotypes, by using A/B genome as the reference, a lot of true homozygous SNPs could be called as heterozygous SNPs due to misalignment. (3) M2 revealed a decent concordance rate (67.61%) of homozygous SNPs and could identify new and true polymorphisms that were not found by the HAPLOSWEEP approach. (4) When using HAPLOSWEEP, the alignment to A/B genome (M4) revealed more homozygous SNPs than alignment to the A + B genome (M5), however, M5 could also identify new and true polymorphisms that were not covered by M4. In summary, none of the pipelines above could cover all possible polymorphisms between the genotypes. However, the best option among the five analysis pipelines was to align the reads to A/B genome followed by HAPLOSWEEP, which can yield the highest amount of homozygous SNPs with a high concordance rate with the SNP array, similar to the rate reported in the recent study (74%) (Clevenger et al., 2018). Alternatively, a better choice would be applying multiple pipelines to get non-redundant SNPs. As an example, methods M2 and M4 may complement each other and would yield more homozygous SNPs if both were applied for analysis.

In this study, we used the concatenated A + B genomes from the diploid wild peanut species (Bertioli et al., 2016) as the reference for SNP calling instead of using the tetraploid genomes recently published (Bertioli et al., 2019; Zhuang et al., 2019). One of our main goals in this study was to compare the SNP calling capability using different pipelines and NGS platforms to discover maximum numbers of SNPs in cultivated peanut. This comparison would be reliable as long as the same reference was used for comparison of different platforms or pipelines and the SNPs between the reference and all reads were filtered out. The diploid and tetraploid genomes were highly similar (Bertioli et al., 2019; Zhuang et al., 2019), thus using either genomes as reference would not change the major findings in this study. Particularly, the *Arachis2* SNP array, the tool used for cross-validation of the SNP callings was designed based on the wild diploid genome and the probes designed for TES were also referred to the diploid genomes. Therefore, in this study, a concatenated A + B genome from wild diploid peanut was used for alignment to achieve a good consistency in comparison.

### Candidate Genomic Regions Controlling Peanut Nodulation

The two sets of sister RILs used in this study were selected at the F_6_ generation, derived from the cross between PI 262090 and UF 487A (Peng et al., 2017a). The Nod+ and Nod− RILs, specifically for E4 and E5, were highly identical. Taking advantage of the nearly isogenic nature between the two pairs of sister RILs with one nodulating and the other non-nodualating, we speculated that polymorphic regions between the sister RILs should harbor any potential candidate genes controlling nodulation. In this study, to identify highly confident homozygous SNPs between the RILs, only the homozygous SNPs polymorphic between PI 262090 and UF 487A as well as between the RILs were included as highly confident SNPs and were placed on the graphical maps. The graphical genotype of these two pairs of RILs allowed us to visualize the polymorphic genome regions harboring candidate genes. The polymorphic regions on the graphical genotype maps could provide guidance for future genetic mapping of nodulation genes in peanut, although these regions were quite big containing a large number of genes since no mapping and fine mapping strategies were applied yet in the current study. We specifically listed out the DEGs involved in nodulation and any orthologs of nodulation related genes as candidates, subsequently obtained a relatively large number of candidates in the genome. These large number of candidate genes was coming from the preliminary comparisons between the two pairs of near-isogenic RILs. Further mapping and fine-mapping strategies should be applied to narrow down and pinpoint the causative genes for non-nodulations in our non-nodulating lines, which will be conducted in a different study.

## Conclusion

Based on the findings from this study, several suggestions were made for future SNP identification studies in peanut. SNPs included in the Axiom *Arachis2* array were mostly discovered from 21 peanut genotypes, which may not be representative enough to cover all the genome polymorphisms. Axiom *Arachis2* array would be a good choice for genotyping populations developed from or related to the genotypes used for the initial SNP discovery. However, if the populations to be genotyped are not related with the initial genotypes for the development of the Axiom *Arachis2* array, then other NGS approaches should be considered. If genes or genomic regions of interest are to be focused, TES should be preferably considered, since the potential candidate regions can be specifically included for SNP identification. Among the SNP calling pipelines to be used for NGS data analysis, the best performing pipeline is to align the reads to A/B genome followed by SNP filtering using HAPLOSWEEP. To identify a larger number of true homozygous SNPs, other pipelines, such as the alignment to A + B genome with traditional SNP filtering, can be combined with HAPLOSWEEP.

## Data Availability Statement

The datasets generated for this study can be found in the NCBI SRP093688, BioProject PRJNA354154, BioSample SAMN06041692–SAMN06041727, and NCBI SRP154150.

## Author Contributions

JW conceived the experiments and secured the funding. ZP performed the experiments. ZP and ZZ analyzed the data and drafted the manuscript. JC and DP helped with data analysis. YC and PO-A provided the SNP array data. All authors read and approved the draft.

## Conflict of Interest

The authors declare that the research was conducted in the absence of any commercial or financial relationships that could be construed as a potential conflict of interest.
